# Postural Strategy in Elderly, Middle-Aged, and Young People during Local Vibratory Stimulation for Proprioceptive Inputs

**DOI:** 10.3390/geriatrics3040093

**Published:** 2018-12-19

**Authors:** Tadashi Ito, Yoshihito Sakai, Kazunori Yamazaki, Reiya Nishio, Yohei Ito, Yoshifumi Morita

**Affiliations:** 1 Three-Dimensional Motion Analysis Room, Aichi Prefectural Mikawa Aoitori Medical and Rehabilitation Center for Developmental Disabilities, Okazaki 444-0002, Japan; 2 School of Design & Architecture, Nagoya City University, Nagoya 464-0083, Japan; 3 Department of Orthopedic Surgery, National Center for Geriatrics and Gerontology, Obu 474-8511, Japan; jsakai@ncgg.go.jp; 4 Faculty of Clinical Engineering, School of Health Sciences, Fujita Health University, Toyoake 470-1192, Japan; ymzkk@fujita-hu.ac.jp; 5 Department of Electrical and Mechanical Engineering, Graduate School of Engineering, Nagoya Institute of Technology, Nagoya 466-8555, Japan; r.nishio.115@nitech.jp (R.N.); y.ito.359@nitech.jp (Y.I.); morita@nitech.ac.jp (Y.M.)

**Keywords:** postural strategy, proprioceptive control, Meissner corpuscle

## Abstract

Proprioceptive input may greatly affect postural stability. However, the proprioceptive postural strategy in elderly, middle-aged, and young people has not been investigated sufficiently. Hence, in this study, we aimed to investigate differences in proprioceptive postural strategies of elderly, middle-aged, and young people. The center of pressure displacement was determined in 23 elderly, 23 middle-aged, and 23 young people during upright stance on a balance board with their eyes closed. Vibratory stimulations at 30, 60, and 240 Hz were applied to the lumbar multifidus (LM) and gastrocnemius (GS) muscles to evaluate the contributions of different proprioceptive signals used in balance control. Compared with middle-aged and young people, elderly people showed a high dependence on postural control of the GS at 30 Hz (*p*-values: Young and elderly: 0.033; middle-aged and elderly: 0.001). Moreover, compared with young people, elderly people were more dependent on postural control of the LM at 240 Hz (*p* = 0.016). There were no significant differences with respect to the GS at 60 and 240 Hz, and with respect to the LM at 30 and 60 Hz between the elderly, young, and middle-aged people. Thus, the postural control strategy of elderly people depends on the GS at 30 Hz.

## 1. Introduction

Postural instability involves pain and declines in the postural strategy, muscle function, and ability of the proprioceptive system [1,2,3]. Postural strategy has been shown to be based on the location rather than being intrinsic to the proprioceptive system [4]. Other studies have reported that elderly people use a more rigid strategy that involves the ankle to cope with postural instability [5]. Moreover, risk factors associated with falls in elderly people include a decline in postural strategy and impairment of the proprioceptive system [6]. Proprioceptive impairment might further lead to instability in postural sway [1,2,3,4,5,6,7]. Therefore, the proprioceptive system is likely to affect postural control. Muscle vibration, which is known to be a strong stimulus for the Meissner corpuscles, muscle spindles, and Vater–Pacini corpuscles, has been used to assess the role of the proprioceptive postural strategy [2,8]. Investigating the role of proprioception under individual stimulation conditions is essential to gain insight into the variability of postural strategies and the possibility of impaired proprioception. Studies have also indicated disturbances of proprioceptive sensory input in younger or elderly people [5,6,7,9]. However, differences in postural stability resulting from the proprioceptive information generated in the trunk and lower limbs in elderly, middle-aged, and young people have not been investigated. Therefore, this study aimed to evaluate the dependence of elderly, middle-aged, and young people on their trunk or lower limbs for proprioceptive postural control.

## 2. Material and Methods

### 2.1. Participants

A cross-sectional study was carried out for over 3 years and 3 months (November 2012 to February 2016). A total of 69 people were recruited for the study, including 23 elderly, 23 middle-aged, and 23 young people. The participants were volunteers from Japan. Participants with the following characteristics were excluded: Neurological disorders, balance disorders, vestibular function disorders, paralysis, ataxia, spinal cord tumor, spinal infection, and history of spinal surgery or joint replacement operation.

Written informed consent was obtained from participants before inclusion in the study. Investigations were conducted according to the principles expressed in the Declaration of Helsinki. The Ethics Committee of the National Center for Geriatrics and Gerontology approved the study (Institutional Review Board approval number: 586).

### 2.2. Measurements

#### Postural Stability Assessment

Postural stability characteristics were measured using a balance board (Wii, Nintendo Co., Ltd., Kyoto, Japan) [8,10,11,12]. Balance board data were acquired using a sampling frequency of 100 Hz and calculated using Matlab (MathWorks, Inc., Natick, MA, USA) with a low-pass filter and a cutoff frequency of 20 Hz. The participants stood barefoot on the balance board with their feet together and their eyes closed. Muscle vibrations were applied bilaterally to the gastrocnemius (GS) and lumbar multifidus (LM) muscles, which respectively stimulated the Meissner corpuscles and muscle spindles, and Vater–Pacini corpuscles. This method was used to evaluate the role of proprioception in postural control [2,5]. The center-of-pressure (COP) of each participant was measured under six conditions in two muscles with three frequencies of vibratory stimulation (0.8 mm of sinusoidal): (1) 30 Hz on GS, (2) 30 Hz on LM, (3) 60 Hz on GS, (4) 60 Hz on LM, (5) 240 Hz on GS, and (6) 240 Hz on LM [2,13]. The mean COP displacement was defined as follows: *Δ*Y = Y (During) − Y (Pre). Further, the COP of the Y–coordinate data was calculated by the root mean square (RMS) values of the COP displacements. The measurement time was 30 s, which was divided into two intervals of 15 s each. Vibratory stimulation was applied to the participants during the last 15 s. We labeled the first 15 s as “Pre” and the last 15 s as “During.” The participants rested on a chair for 60 s between measurements. Assessment measures were performed by an experienced doctor and a physiotherapist.

### 2.3. Statistical Analysis

Data were analyzed using the Statistical Package for Social Sciences version 19.0 for Windows (SPSS Inc., Chicago, IL, USA). *P* < 0.05 was considered statistically significant. Differences between the three groups and the postural strategy of the muscle vibration trials were analyzed using one-way analysis of variance (ANOVA). Variables for significance were compared using multiple comparison analysis with Bonferroni correction.

## 3. Results

**Figure 1:**
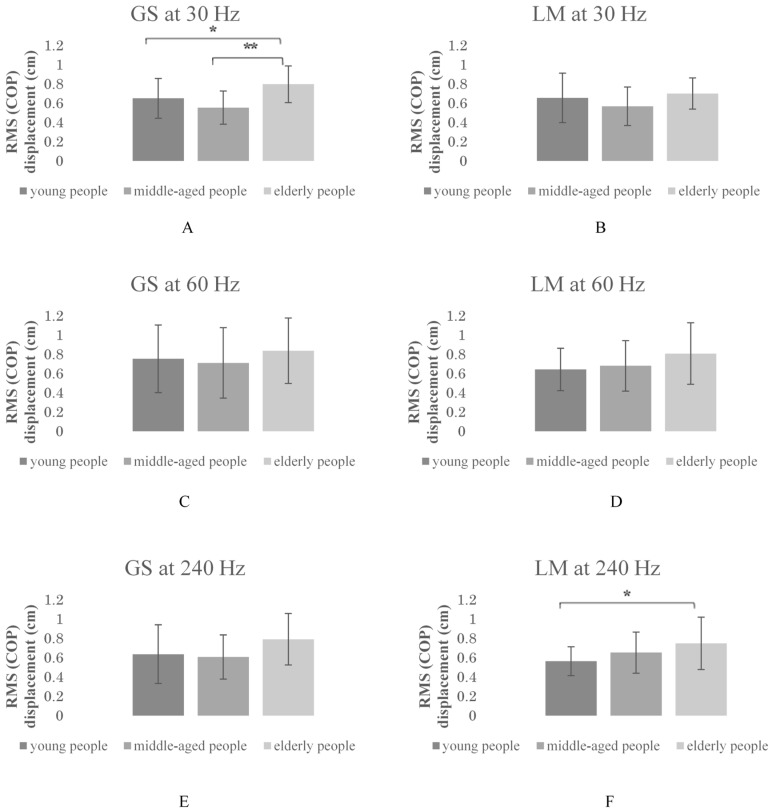
Root mean square (RMS) values of the center of pressure (COP) displacement for the trials on the balance board. GS: gastrocnemius; LM: lumbar multifidus. * *p* < 0.05; ** *p* < 0.001; multiple comparison analysis using Bonferroni correction.

Table 1 shows the baseline characteristics of the study participants. Figure 1 displays the mean RMS values of COP displacements for the six postural stability trials for each group. As a result of the one-way ANOVA, the significance between groups and muscle vibration trials (30 and 60 Hz on GS and 240 Hz on LM) was determined (Table 2). The multiple comparison analysis in the Bonferroni correction showed that compared with middle-aged and young people, elderly people were dependent on the postural control of the GS at 30 Hz (Figure 1A and Table 3). Further, compared with young people, elderly people were dependent on the postural control of the LM at 240 Hz (Figure 1F and Table 3). In contrast, the postural control of the GS at 60 and 240 Hz and LM at 30 and 60 Hz did not have a significant influence on the elderly compared to young and middle-aged people.

## 4. Discussion

Our main finding was that elderly people relied on proprioceptive signals from the Meissner corpuscles of the GS and Vater–Pacini corpuscles from the LM during postural control. These results indicate that the postural strategy adopted by elderly individuals is dependent on the proprioceptive input via the Meissner corpuscles of the GS and Vater–Pacini corpuscles of the LM. Previous studies have already demonstrated that elderly people with low back pain (LBP) relied on lower-limb (30 Hz) and trunk-based strategies (240 Hz) for postural control during vibratory stimulation [13].

The higher COP displacement values in elderly people, compared with middle-aged and young people, during vibratory stimulations of 30 Hz indicate that elderly people rely on lower-limb-muscle proprioceptive inputs. Previous studies have reported that the number of Meissner corpuscles reduces with age [14]. Moreover, information generated by the Meissner corpuscles from the lower limbs affects postural control.

Furthermore, significant differences in COP displacements at the LM with 240 Hz stimulation were found between young and elderly people. These results are in disagreement with those of previous studies where no significant differences in postural sways could be found between people with LBP and healthy people during quiet standing conditions [15,16,17]. Brumagne et al. reported that a trunk stiffening strategy or ankle strategy could be sufficient for controlling posture in simple postural conditions, leading to tighter control of COP and smaller postural sways [17]. Moreover, healthy people normally maintain postural stability using a multisegmental control strategy [18,19]. Accordingly, the middle-aged and young people significantly mutually upweighted the proprioceptive signals from the Meissner corpuscles, muscle spindles, and Vater–Pacini corpuscles, leading to a tighter control of the COP than that from the GS and LM to control postural balance. In contrast, the postural strategy of elderly people depends on the Meissner corpuscles of the GS and Vater–Pacini corpuscles of the LM to achieve postural stability. However, no significant differences in COP displacement with 240 Hz stimulation at the LM were found between the middle-aged and elderly people. Therefore, elderly people may depend more on the Meissner corpuscles of their lower limbs than on the Vater–Pacini corpuscles of the LM. In contrast, the proprioception of postural maintenance has been reported to be in the range of 25–30 Hz for the lower-limb and trunk muscles [20]. In addition, use in postural control of the tactile information from the GS has been suggested [21].

These findings clearly show the differences in proprioception with respect to age, suggesting that aging may influence the proprioceptive inputs derived from the Meissner corpuscles of the GS. Hence, assessing the effect of proprioceptive input during postural control testing could provide insights into the role of proprioception in rehabilitation programs directed towards elderly individuals. Furthermore, based on the results of the muscles vibratory stimulus, a proprioceptive activation program for the GS and LM may be utilized to optimize the treatment of proprioception.

Nevertheless, this study has some limitations. First, only community-dwelling elderly, middle-aged, and young people were investigated. Second, the assessment of visual control was limited to the experiments being performed with the participant’s eyes closed; blackout glasses were not used. Additional study is needed to determine whether age-associated difference in postural strategy of proprioceptive sensitivity (Meissner corpuscle) is caused by declines in physical performance or other characteristics.

## 5. Conclusions

No previous research has investigated the differences in proprioceptive postural strategies with low and high frequencies of vibration in young people, middle-aged, and elderly people. Elderly people exhibit varying dependence on the proprioceptive postural strategies of the GS (Meissner corpuscle) because of aging. Increased reliance on GS proprioceptive input (Meissner corpuscle) during standing on a stable surface slightly increases the postural instability in future elderly people. Objective assessment of proprioceptive postural control strategy might be important in identifying limitations in proprioceptive inputs or function.

## Figures and Tables

**Table 1:** Demographic characteristics of the participants.

Variables	Young (n = 23)	Middle-Aged (n = 23)	Elderly (n = 23)
Age (years)	21.7 ± 1.3	46.0 ± 3.1	72.1 ± 4.8
Male/female	11/12	11/12	14/9
Height (cm)	165.5 ± 6.4	164.9 ± 8.1	159.0 ± 9.1
Weight (kg)	55.8 ± 8.1	60.1 ± 11.0	63.6 ± 12.3
BMI (kg/m^2^)	20.3 ± 2.5	22.0 ± 2.8	22.5 ± 3.7

BMI, body mass index; SD: standard deviation. Data are presented as the mean ± SD.

**Table 2:** Results of the one-way analysis of variance.

Variables	Sum of Squares	Degrees of Freedom	Mean Squares	F-Value	*p*-Value
GS at 30 Hz	0.683	2	0.342	9.413	0.001
GS at 60 Hz	0.189	2	0.094	0.806	0.451
GS at 240 Hz	0.448	2	0.224	3.128	0.05
LM at 30 Hz	0.209	2	0.104	2.36	0.102
LM at 60 Hz	0.346	2	0.173	2.359	0.102
LM at 240 Hz	0.391	2	0.195	4.144	0.02

GS, gastrocnemius; LM, lumbar multifidus.

**Table 3:** Mean COP displacements during the vibration trials for each group when standing on a balance board.

Variables	Young (n = 23)	Middle-aged (n = 23)	Elderly (n = 23)	*p*-Value
GS at 30 Hz	0.65 ± 0.21	0.55 ± 0.17	0.80 ± 0.19	Young and middle-aged: 0.284 Young and elderly: 0.033 Middle-aged and elderly: 0.001
GS at 240 Hz	0.64 ± 0.30	0.61 ± 0.23	0.79 ± 0.27	Young and middle-aged: 1.00 Young and elderly: 0.162 Middle-aged and elderly: 0.07
LM at 240 Hz	0.56 ± 0.15	0.65 ± 0.21	0.75 ± 0.27	Young and middle-aged: 0.051 Young and elderly: 0.016 Middle-aged and elderly: 0.422

GS, gastrocnemius; LM, lumbar multifidus; SD, standard deviation. Data are presented as mean ± SD. Multiple comparison analysis: Bonferroni correction.
